# Personal Space Regulation in Childhood Autism Spectrum Disorders

**DOI:** 10.1371/journal.pone.0074959

**Published:** 2013-09-23

**Authors:** Erica Gessaroli, Erica Santelli, Giuseppe di Pellegrino, Francesca Frassinetti

**Affiliations:** 1 Department of Psychology, University of Bologna, Bologna, Italy; 2 Fondazione Salvatore Maugeri, Clinica del Lavoro e della Riabilitazione, Istituto di Ricovero e Cura a Carattere Scientifico, Mantova, Castel Goffredo, Italy; 3 Centro Autismo, Reggio Emilia, Italy; 4 Center for Studies and Research in Cognitive Neuroscience, Cesena, Italy; University of Western Brittany, France

## Abstract

People appropriately adjust the distance between themselves and others during social interaction, and they may feel discomfort and move away when another person intrudes on their personal space. In the present study, we investigated personal space in children with persistent difficulties in the domain of social behavior, such as children with autism spectrum disorders (ASD), and in children with typical development (TD). The stop-distance paradigm was used to derive estimates of interpersonal distance, before and after a brief interaction with an unfamiliar adult confederate. The results showed that ASD children felt comfortable at a greater distance compared to TD children. Moreover, personal space shrunk after interaction with the confederate in TD children, but it failed to do so in ASD children. These findings reveal that autism deeply affects the regulation of personal space, influencing both its size and flexibility.

## Introduction

Personal space is the area individuals maintain around themselves and into which intrusion by others may cause discomfort or even anxiety. People closely monitor and appropriately regulate their interpersonal space to obtain a comfortable distance of interaction with others [1-4]. When personal space is violated, the person may move away to reinstate the margin of safety. Thus, personal space is fundamentally a protective space, a zone of safety surrounding the body [5].

A number of studies have shown that the size of the personal space varies depending on social context. A person who is placed in a potentially threatening context will have an expanded personal pace; a person in friendly company will have a reduced personal space [4,6]. Moreover, the size of interpersonal space can change as a function of different factors, including gender [7], age [8], infant–caregiver attachment [9,10] and familiarity between interacting parties [11,12]. Studies have also documented that psychiatric [13], neurological [14], and developmental disorders [15] can interfere with the regulation of personal space.

More recently, Kennedy and coworkers [14] described the regulation of interpersonal distance in a patient (SM) with bilateral amygdala damage. In their experiment, the authors asked SM to indicate the position at which she felt most comfortable as an experimenter approached her, or she approached the experimenter. SM showed a substantially reduced personal space compared to comparison subjects. A questionnaire, in which the patient rated her level of comfort/discomfort standing to different distances from the experimenter, put in evidence that SM was perfectly comfortable also at a nose-to-nose distance with the experimenter. These findings revealed that bilateral damage to the amygdala results in no detectable personal space boundary and an abnormally small interpersonal distance preference, thereby suggesting that this brain structure is part of the neural substrate regulating the distance between individuals. Moreover, neuroimaging data from healthy subjects in this same study [14] showed a greater activation of the amygdala when participants knew that an experimenter was maintaining a close distance to them, compared to when they knew that an experimenter was maintaining a far distance. This conclusion is supported by the results of non-human primate studies, revealing that monkeys with bilateral amygdalar damage preferred to stay in closer proximity to other monkeys or people compared to monkeys without lesion [16-18].

Because personal space represents the space of interaction and communication with others, it is critical to study this space in subjects with everyday difficulties in social and emotional behavior, such as patients with autism spectrum disorders (ASD). Autism is a neurodevelopmental disorder characterized by marked and enduring deficits of interpersonal interaction, including behavioral avoidance and unresponsiveness [19-23], and failure to spontaneously interact with people [24,25]. Moreover, it has been proposed that dysfunction of the amygdala may be responsible, at least in part, for the impairment of social and emotional functioning that is a core feature of autism [26-29]. However, relatively little is known about the way in which autistic individuals regulate the physical distance from other people during social interactions. Although anecdotal observations and some meager evidence [30] suggest that the ability to reliably regulate one’s distance from other people may be impaired in ASD, interpersonal distance has never been directly measured in individuals with autism in a laboratory test.

In the present study, our primary aim was to provide a direct measure of the personal space of children with typical development (TD) and children with an impairment in social approach, such as autism (ASD). The second aim was to investigate the modulation of personal space by a brief social interaction with an unfamiliar other in these two populations of children. To this end, we measured personal space using a modified version of the stop-distance procedure [31-33]. This paradigm represents one of the most frequently used measure of personal space regulation, allowing reliable estimates of preferred interpersonal distance under varied conditions and repeated measures (for reviews, see [8,33]). In our experiment, personal space was measured as the distance at which children felt most comfortable as an unfamiliar adult confederate approached them or they approached the confederate. Each participant was tested twice, i.e., before and after a break during which participant interacted with the confederate.

Prior research has suggested that an excessively functioning amygdala may account for abnormal fears and enhanced anxiety in autistic children, leading to impaired social interactions and avoidant behaviors in these patients [34-39]. Accordingly, we hypothesized that ASD children, due to increased fear and hyperarousal following personal space violations, would fail to reliably and flexibly regulate personal space, thereby maintaining a farther and rigid distance from others. As a consequence, we predicted that interpersonal distance would be larger in ASDs than in TD children and it should be modulated by a brief social interaction in TD but not in ASDs children.

## Methods

### Ethics statement

The study involved children with autism spectrum disorders and children with typical development in a behavioral experiment. Subjects’ parents gave written informed consent to their children’s participation in the study, which was approved by the ethics committee of the Centro Autismo, Ausl, Reggio Emilia, where the experiment was performed, and by the ethics committee of the Department of Psychology of the University of Bologna. The experiment was conducted according to the ethical guidelines of the Declaration of Helsinki.

### Participants

Fifteen male children with autism spectrum disorders (ASD) participated in the study. The autistic children were recruited through referrals from a center for children with ASD (Reggio Emilia, Italy). They will hereby be designated as the group of individuals with ASD. All had received a formal diagnosis of an ASD by an independent clinician, according to the standard Diagnostic and Statistical Manual of Mental Disorders-IV criteria [40] and all were high functioning. The diagnosis was confirmed using the Autism Diagnostic Observation Schedule-Generic (ADOS-G) scale [41], given by a trained clinical psychologist. ASD children had all fluent language abilities. They had no other diagnosed neurological (e.g. cerebral palsy or epilepsy) or medical disorders, and none of them were taking antipsychotic drugs at the time of testing.

We compared the ASD children to 23 male children with typical development (henceforth TD children). TD children were recruited in local schools and were free of current or past psychiatric or neurological illness as determined by history.

ASD and TD groups did not differ with respect to both mental (TD = 9.17 years, sd = 1.03 years; ASD= 9.07 years, sd =2.43 years; [F(1,36) = 0.05; p = .85]), and chronological age (TD = 9.56 years, sd = 1.73 years; ASD = 9.73 years, sd =2.37 years; [F(1,36) = .06; p = .80]; see Table 1). The mental age was calculated by using the formula (chronological age X IQ/100). The Total IQ scores were measured with the WISC-III, submitted to children in a session different from the experimental session.

**Table 1:** Subject Demographics for Children Participating in the Study.

	**ASD Group** (**N=15**) (Mean/SD)	**TD Group** (**N=23**) (Mean/SD)
Chronological Age	9.73 (+/- 2.37)	9.56 (+/- 1.73)
Mental Age	9.07 (+/- 2.43)	9.17 (+/- 1.03)
Full Scale IQ	92.73 (+/- 16.08)	97.61 (+/- 10.76)
ADOS (Full Scale)	15.6 (+/- 3.37)	NA
ADOS (Social interaction)	8 (+/- 2.24)	NA
ADOS (Communication)	5.8 (+/- 3.12)	NA
ADOS (Imagination)	1.2 (+/- 0.77)	NA
ADOS (Behaviors)	1.67 (+/- 1.72)	NA
Diagnosis	9 (F84.9) 6 (F84.0)	NA

IQ assessed with Wechsler Intelligence Scale for Children–Third Edition (WISC-III) or Wechsler Abbreviated Scale of Intelligence (WASI)

### Procedure

We applied an adapted version of the stop-distance paradigm used by Kennedy et al. [14]. All participants were tested in the same room (7 x 4 m) by one experimenter and one confederate. The role of the experimenter and confederate was taken in turns. Care was taken to ensure that the experimental setup remained identical across participants.

**Figure 1:**
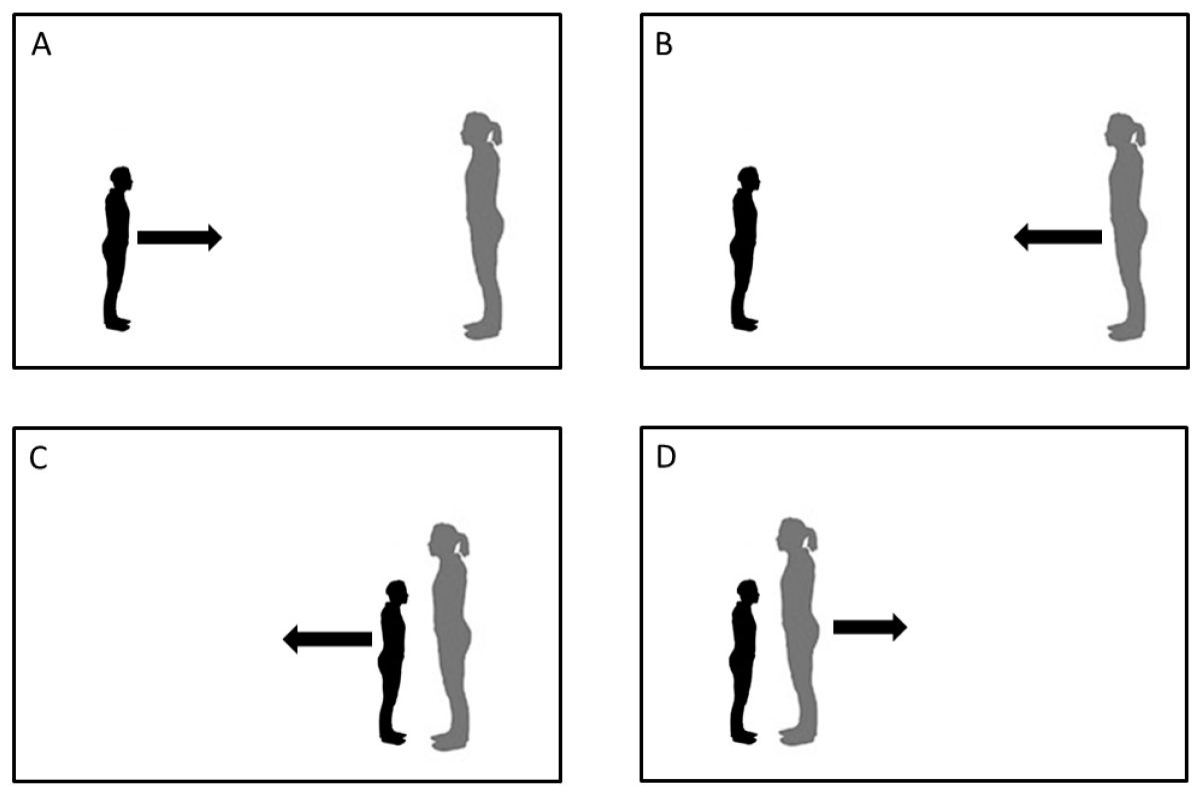
Experimental procedure. In the first condition (A) the participant approached the confederate starting from a far distance (5 m). In the second condition (B) the confederate approached the participant starting from a far distance (5 m). In the third condition (C) the participant moved away from the confederate starting from a close distance (face to face). In the fourth condition (D) the confederate moved away from the participant starting from a close distance (face to face).

Testing began with a participant positioned at a fixed location in the room and the confederate standing, facing the participant from a far starting position (five meters), or from a close starting position (30 cm). In half of the trials, the female confederate was always the one moving, at a natural gait either toward (i.e., far starting position) or away (i.e., close starting position) from the participant. In the other half of the trials, the participant was always the one moving, either approaching or withdrawing from the confederate (see Figure 1). Participants were instructed to tell the confederate to stop at their preferred distance (i.e., the distance between themselves and the confederate at which they felt most comfortable), in the trials when the confederate was moving, and chose their ideal interpersonal distance in the trials when they were moving. During the approach/withdrawal movement, the confederate made no eye contact, maintained a neutral facial expression, and never touched the participant. The interpersonal distance was measured with a digital laser measurer (Agatec, model DM100, error ± .003m), as the distance between the confederate’ toes and the participant’s toes.

The same procedure was repeated twice, before and after a 10-minute time interval. During the time interval, the confederate invited the subject to seat down on a cushion placed in the same room and to read together an illustrated book chosen by the participant. The children could choose one illustrated book among fifteen different ones, which had been suggested by their teachers or psychologists as being particularly interesting for each child. During the interaction, the confederate read the book and asked three questions concerning the content of the book, while each participant was invited to make comments and ask questions to the confederate. In order to measure the amount of this social interaction, the experimenter assigned a score between 0 and 3 to each of three behaviors: i) the child’s ability to answer to the confederate’s questions, ii) the child’s ability to make comments about the book, and iii) the child’s ability to ask questions to the confederate. These three ratings were averaged together to obtain an index of social interaction.

Before starting the experiment all participants received an explanation of the task and had four practice trials with the experimenter. Then the confederate was introduced.

To sum up, we run a 2x2x2 design with starting position (close and far), person moving (confederate and participant) and session (before and after social interaction) as factors. Each cell of the experimental design comprised 3 trials, thus yielding a total of 24 completely randomized trials.

## Results

The effect of social interaction on personal space regulation was verified in children with TD and in children with ASD by comparing the interpersonal distance in the two groups before and after the interaction with the adult confederate. To this aim, a mixed-design analysis of variance (ANOVA) was conducted on the measure of interpersonal distance expressed in mm, with group (ASD and TD) as a between-subject variable, and session (before and after social interaction) as a within-subject variable. For the purpose of this analysis, data were collapsed across person moving (confederate and participant), and starting position (close and far) condition.

**Figure 2:**
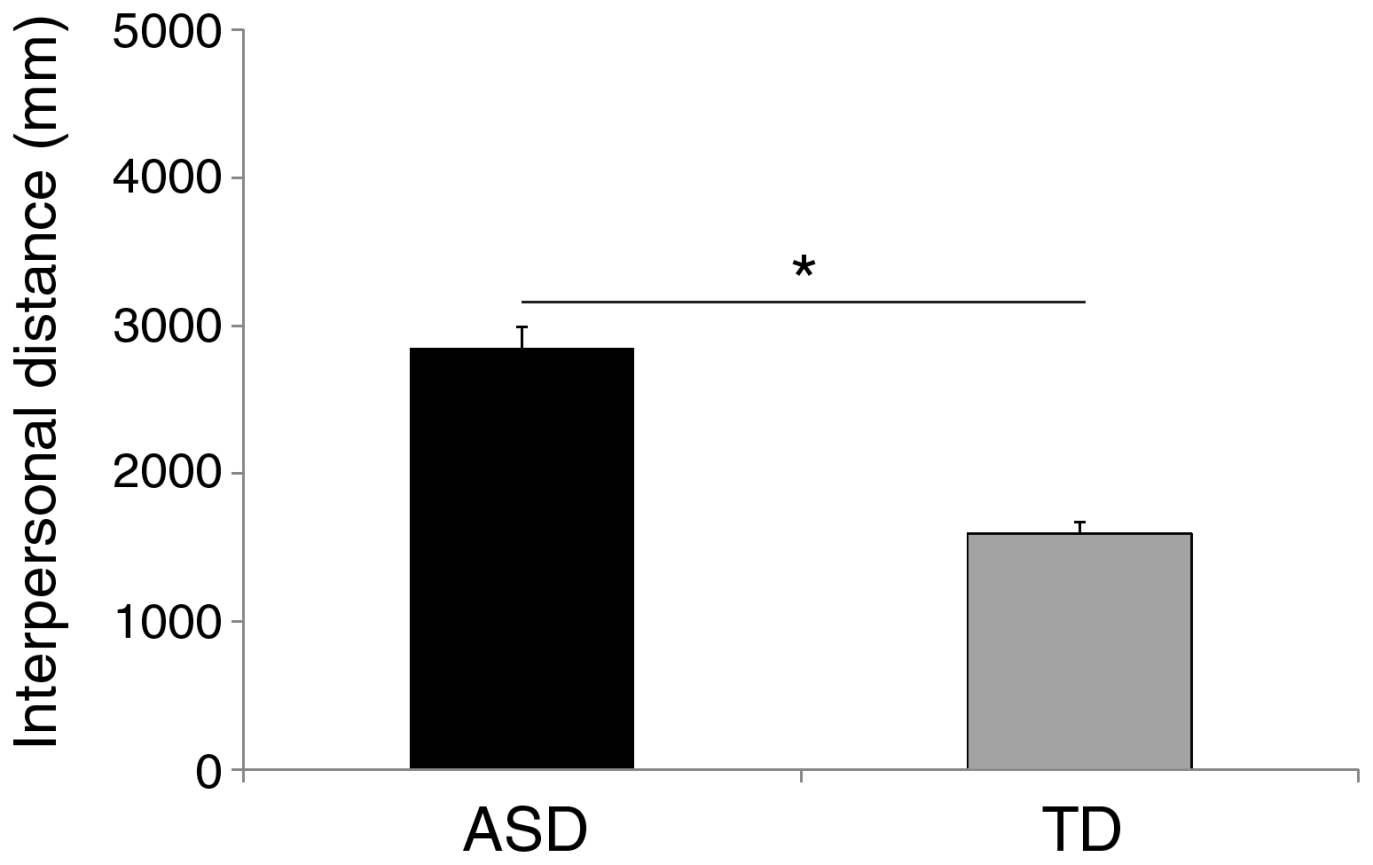
Mean preferred distance from the confederate in children with autism spectrum disorders (ASD), and children with typical development (TD). Asterisk indicates significant comparison (p < 0.05). Error bars denote s.e.m.

The variable group was significant [F(1,36) = 14.84; p < .0001; η_p_^2^= .292], revealing that the interpersonal distance was larger in ASD children than in TD children (2850 mm *vs.* 1595 mm, respectively; see Figure 2). There was also a marginally significant effect of session [F(1,36) = 3.80; p = .06; η_p_^2^ = .096], showing that interpersonal distance was larger before (2175 mm) than after (2022 mm) the social interaction.

**Figure 3:**
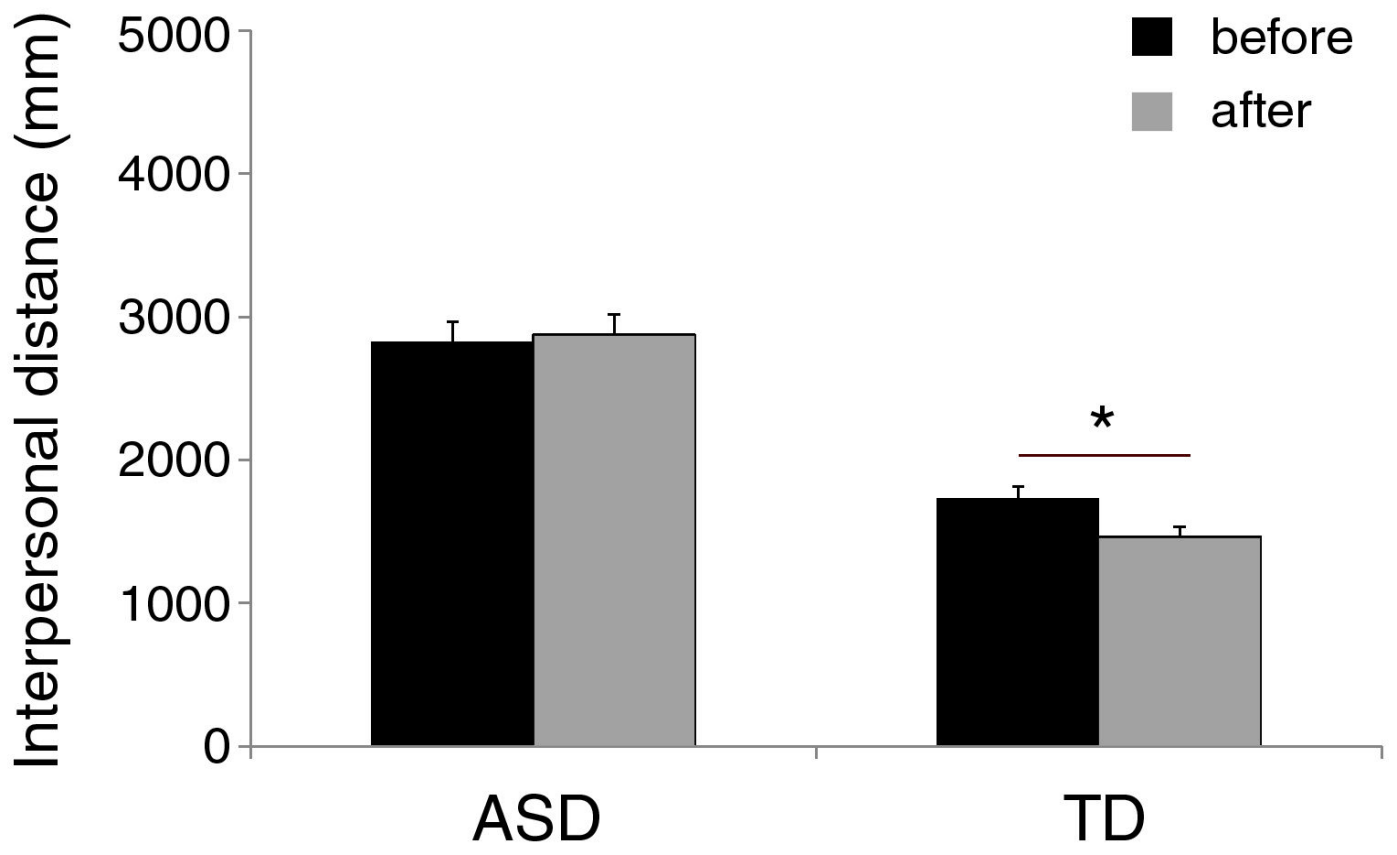
Mean preferred distance from the confederate before and after a brief social interaction in children with typical development (TD), and children with Autism Spectrum Disorder (ASD). Asterisk indicates significant comparison (p < 0.05). Error bars denote s.e.m.

Critically, the main effects were qualified by a significant group X session interaction [F(1,36) = 7.73; p < .01; η_p_^2^ = .177]. Indeed, post-hoc analysis showed that the social interaction between participant and confederate during the interval did not modulate personal space in ASD children (before = 2826 mm, after = 2874 mm, p = .95), whereas it modulated personal space in TD children, reducing the distance after (1461 mm), as compared to before (1730 mm), the social interaction (p < .003; see Figure 3). Moreover, interpersonal distance was larger in ASD children than TD children both before and after social interaction (p < .0002 for both comparisons).

For completeness, we also run an additional ANOVA that included all the variables, and specifically group (ASD and TD) as a between-subject variable, and person moving (confederate and participant), starting position (close and far), and session (before and after social-interaction) as within-subject variables. As before, this second ANOVA demonstrated that the variable group [F(1,36) = 14.84; p < .0001; η_p_^2^ = .292], session [F(1,36) = 3.80; p = .06; η_p_^2^ = .096], and the group X session interaction [F(1,36) = 7.73; p < .01; η_p_^2^ = .177] were significant. We also found that the interaction between group, person moving, and starting-position was significant [F(1,36) = 8.24; p < .006; η_p_^2^ = .186]. Post-hoc analysis of this three-way interaction showed that, in ASD children, the interpersonal distance was significantly larger when the participant moved away from (3376 mm) rather than toward (2413 mm) the confederate (p < .0001). By contrast, this difference was not significant when the confederate moved away or approached the ASD participant (when starting close = 2630 mm; when starting far = 2979 mm, p = .26).

In TD children, this difference was not significant neither when the participant moved away or approached the confederate (when starting close = 1781 mm; when starting far = 1417 mm), nor when the confederate moved away or approached the participant (close starting position = 1.493 mm; far starting position = 1.688 mm; all p > .05). Note, however, that the interpersonal distance remained larger in ASD children than in TD children, regardless of person moving (confederate or participant), or starting position (close or far) (p < .001 in all comparison).

The lack of modulation of interpersonal distance may depend on poor or reduced social interaction with the confederate in ASD compared to TD children, and not on a deficit of personal space regulation. To explore this possibility, an index of social interaction, ranging form 0 to 3, was computed for each participant by averaging together the scores assigned by the experimenter to three behaviors observed during the child-confederate interaction (see Method). Although the social interaction index of the ASD group was somewhat lower than control group (2 and 2.3, in ASD and TD children, respectively), the analysis did not reveal a significant main effect of group, [F(1,36) = 0.71; p < .4]. Nevertheless, to ensure that our findings were not driven by subtle group differences in the amount of social interaction with confederate, the main ANOVA was repeated with the social interaction index as covariate. The previously significant group X session interaction remained significant, [F(1,35) = 6.8; p < .01; η_p_^2^ = .16]. As a further control analysis, we ranked ASD participants based on the index of social interaction and divided participants into good-interaction (ASDgi, n = 7) and poor-interaction (ASDpi, n = 8) groups thorough a median split. Finally, an ANOVA was performed on the measure of the personal space difference (interpersonal distance after interaction – before interaction), with group (ASDpi, ASDgi) as between-subject variable. The variable group was not significant [F(1,13) = 1.01; p = 0.33]. Overall, these data suggest that the lack of modulation of interpersonal distance were not due to reduced social interaction with the confederate during the interval in ASD compared to TD children.

### Control experiment

Our data suggest that social interaction influences personal space in TD and not in ASD children. However, to ensure that this effect in TD children was not simply due to the time interval between the first and the second measure, or to a familiarization with the task or with the confederate, rather than to the effect of social interaction between the confederate and the subject, an additional control group of 23 age-matched TD children (TD-C) was tested.

The TD-C group was submitted to the same procedure previously described with the only difference that during the time interval participant and confederate did not read a book together, but the subject read a book by himself, while the confederate was busy doing something else in the same room. If the reduction of the personal distance observed in TD children was due to time interval *per se*, then it should be found both in the TD and in the TD-C group. By contrast, if the reduction of the interpersonal distance in TD was due to the interaction between confederate and subject during the time interval, then it should be found only in TD but not in TD-C group. TD-C children and TD were compared by an ANOVA with group (with and without social interaction) as a between-subject variable, and with session (before and after time-interval), as a within-subject variable.

**Figure 4:**
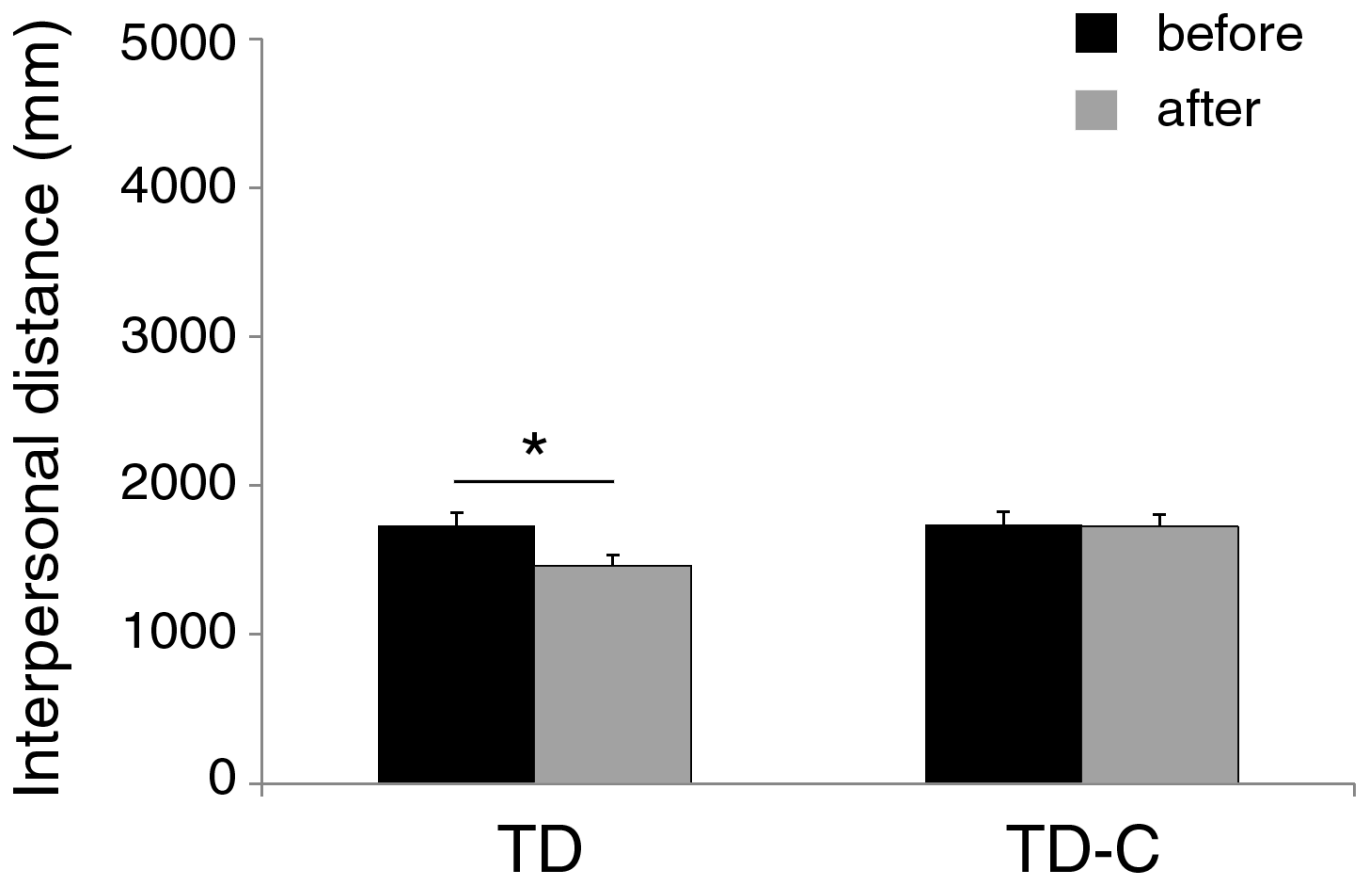
Mean preferred distance from the confederate in two groups of children with typical development before and after a time interval during which the confederate interacted (TD group), or not (TD-C group), with the participant. Asterisk indicates significant comparison (p < 0.05). Error bars denote s.e.m.

The variable session was significant [F(1,44) = 11.49; p < .001; η_p_^2^ = .21], showing that interpersonal distance was smaller after (1592 mm) than before (1733 mm) the time interval. In line with the hypothesis, the group X session interaction was significant [F(1,44) = 9.48; p < .001; η_p_^2^ = .18]. Post-hoc comparisons (Tukey for equal N) showed a reduction of personal space in the group with social interaction (TD group, before = 1730 mm, after =1460 mm, p < .0002), but not in the group without social interaction (TD-C group, before = 1735 mm, after = 1722 mm, p = .97). Moreover, the personal space was significantly different between the two groups after (p < .0001) but not before (p = .92) time interval (Figure 4).

## Discussion

In this study, we investigated personal space regulation in children with typical development (TD) and in children with high-functioning autism, before and after a brief interaction with an unfamiliar adult confederate. While previous anecdotal observations have suggested that ASD children have some difficulties in appropriately regulating one’s distance from other people during social interactions, the empirical evidence supporting this claim has been conspicuously lacking. Here, a stop-distance procedure was used to derive measures reflecting tolerance of, and reactiveness to, spatial-intrusion in ASD and TD children. We provide new evidence that personal space regulation is impaired in high-functioning ASD children. Specifically, we found that ASD children are less tolerant of close proximity to an unfamiliar adult and prefer farther interpersonal distance compared to TD children. Moreover, results showed that interpersonal distance is larger in autistic children when they *move away* from, rather than toward, the confederate, suggesting that these children feel more uncomfortable and react (i.e., step away) more strongly following personal space violations (i.e. close starting position) than TD children.

A critical finding of the present study concerns the modulation of a brief social interaction on personal space regulation. Previous studies focused on the effects on personal space of long-lasting interaction, such as infant-caregiver attachment [9,10] and familiarity between interacting partners [11,12]. Here, we report that a transitory social interaction with an unknown adult results in a rapid, on-line adjustment of the interpersonal distance in TD children, indicating that personal space regulation exhibits dynamic properties and higher flexibility that may facilitate social interactions and communication in normal individuals. Such interpersonal distance changes were not simply due to the effects of time interval between the first and the second interpersonal distance measurement, or to participants’ familiarization with the stop-distance procedure or the adult confederate, as demonstrated by the results of a control experiment. Indeed, when during the interval between first and second stop-distance procedure normally developing children read a book alone without interacting with the confederate, no change in the size of personal space was observed. Critically, ASD individuals failed to display changes of social distance in response to a brief social interaction, suggesting a marked inflexibility of personal space in this condition.

In the past few years, a distinction has arisen between flexibility and permeability of personal space. Permeability refers to the ease with which personal space is penetrated or intruded upon, irrespective of its current size or shape, while flexibility refers to situationally induced changes in the size and shape of personal space [42,43]. Our data suggest that, in autistic children, personal space is altered both in permeability, since it is larger in ASD then in TD children, and in flexibility, since it is not reduced by a social interaction with the confederate. We propose that the impairment in flexibility and permeability of personal space in ASD children reflects overarousal and enhanced fear induced by others intruding their social space.

A previous lesion and neuroimaging study in humans suggested that the amygdala plays a key role in underpinning personal space regulation [14], either by triggering innate emotional reactions in response to personal space violations, or learning the association between close distance and aversive outcomes. Linking these previous results to the present findings, we suggest that reduced tolerance of physical closeness with a stranger and lack of flexibility of personal space in ASD children may result from impairment of an amygdala-based mechanism. This hypothesis is supported by several data. Recent studies indicate that the amygdala is enlarged in children with autism [44] and could contribute to the abnormalities of fear and anxiety that appear to be a common feature of autism. An excessively functioning amygdala may account for the increased autonomic responses in autistic children (e.g., [34,37], but see also [45-47] for different results) leading to withdrawal from social interactions [37]. Moreover, functional imaging in older children and adults with autism provide evidence of an abnormal pattern of amygdala activation in response to social stimuli [39,48-50]. This is further confirmed by findings in an animal model of autism, in which rats exposed to valproic acid exhibit autism-like symptoms associated with enhanced anxiety and fear processing in the amygdale [38]. Finally, recent evidence indicates that oxytocin, a neuropeptide known to reduce activity in the amygdala, thereby resulting in decreased fear responses [51], can modulate social distance in interacting partners [52], and improve social interactions in ASD individuals [53].

Several prior observations are in keeping with the present findings. Employing the naturalistic observation method, Rogers and Fine [54] compared the personal distance behaviors of an autistic and asymbiotic psychotic child during play therapy. The autistic child maintained a greater personal distance from the therapist compared to the symbiotic child. Moreover, Parson and colleagues [55] compared the ability to understand and use some virtual environments, such as a Virtual Cafè, in a social congruent way, in ASD participants of 13-18 years of age and in age-matched control participants. The results showed that the majority of autistic subjects seemed to have a basic understanding of the virtual environment as a representation of reality, but when participant’s ability relative to some social norms was judged by naïve rates, autistics were more likely to be judged as bumping into, or walking between, other people in the virtual scene, compared to their paired matches. The authors suggested that understanding personal space is impaired in autism. More recently, Kennedy and colleagues [30], analyzing parent- and teacher-report questionnaire ratings, concluded that ASDs children are less aware of social distance than their unaffected siblings, showing significantly higher levels of interpersonal distance violations than controls. Overall, these previous findings are consistent with the present results, supporting the general conclusion that interpersonal distance regulation is impaired in autism. Still, results of increased violations of personal space in autistic individuals reported both by Parsons et al. [55] and Kennedy et al. [30] studies are not in accordance with behavioral patterns observed in the present study, in which ASD children exhibit large interpersonal distance preference. However, several methodological differences between previous studies and the present one may account for the seemingly discrepant results. For instance, Parson and colleagues [55] study differed from ours in that they used virtual figures and scenes to probe personal space. It is possible that participants in the ASD group bumped into the people in the virtual environment because they have difficulty understanding the virtual environment as representations of reality. Likewise, Kennedy and colleagues [30] analyzed questionnaire-based data and did not provide a direct and controlled assessment of personal space in ASD children. Thus, interpersonal distance measures and paradigms remain to be systematically compared in autism.

Two potential limitations of this study deserve mention. First, our suggestion that increased fear and hyperarousal following personal space violations would result in larger interpersonal boundaries in ASD compared to TD children remains speculative. Physiological reactions, such as skin conductance responses and heart rate, and subjective ratings of experience may provide potential measures of affect. While the present behavioural data support the claim that personal space regulation is impaired in autism, they cannot directly ascertain the role of affective processes in driving the difficulty with social space in ASD children. Second, as interpersonal distance in the present study was assessed in a controlled experimental setting, we should be cautious about generalizing the findings to other, more ecological settings. Discrepancy between the current and previous findings [30,55] may reflect differences across various settings.

To conclude, discomfort and fear of physical closeness with a social partner may be one of the most salient factors in regulating interpersonal distance during social interaction [56]. Here, we report that ASD children maintain a farther and rigid distance from unfamiliar others than do TD individuals, suggesting that they are less tolerant and more reactive to violations of personal space. We suggest that these effects could arise in part through enhanced, rather than reduced, amygdala functioning in childhood autism spectrum disorders. A better characterization of the mechanisms involved in abnormal personal space regulation in ASD children may lead to an improved understanding of how ASD develops and how to intervene to improve social functioning.
